# Lifestyle interventions for overweight and obese pregnant women to improve pregnancy outcome: systematic review and meta-analysis

**DOI:** 10.1186/1741-7015-10-47

**Published:** 2012-05-10

**Authors:** Eugene Oteng-Ntim, Rajesh Varma, Helen Croker, Lucilla Poston, Pat Doyle

**Affiliations:** 1 Department of Women's Health, Guy's and St Thomas' NHS Foundation Trust (King's Health Partners), St Thomas' Hospital, Westminster Bridge Road, London, SE 1 7EH, UK; 2 London School of Hygiene and Tropical Medicine, Keppel Street, London, WC1E 7HT, UK; 3 School of Medicine, King's College London, Strand, London, WC2R 2LS, UK; 4 Department of Epidemiology and Public Health, University College London, Gower Street, London WC1E 6BT, UK

## Abstract

**Background:**

Overweight and obesity pose a big challenge to pregnancy as they are associated with adverse maternal and perinatal outcome. Evidence of lifestyle intervention resulting in improved pregnancy outcome is conflicting. Hence the objective of this study is to determine the efficacy of antenatal dietary, activity, behaviour or lifestyle interventions in overweight and obese pregnant women to improve maternal and perinatal outcomes.

**Methods:**

A systematic review and meta-analyses of randomised and non-randomised clinical trials following prior registration (CRD420111122 http://www.crd.york.ac.uk/PROSPERO) and PRISMA guidelines was employed. A search of the Cochrane Library, EMBASE, MEDLINE, CINAHL, Maternity and Infant care and eight other databases for studies published prior to January 2012 was undertaken. Electronic literature searches, study selection, methodology and quality appraisal were performed independently by two authors. Methodological quality of the studies was assessed according to Cochrane risk of bias tool. All appropriate randomised and non-randomised clinical trials were included while exclusions consisted of interventions in pregnant women who were not overweight or obese, had pre-existing diabetes or polycystic ovarian syndrome, and systematic reviews. Maternal outcome measures, including maternal gestational weight gain, gestational diabetes and Caesarean section, were documented. Fetal outcomes, including large for gestational age and macrosomia (birth weight > 4 kg), were also documented.

**Results:**

Thirteen randomised and six non-randomised clinical trials were identified and included in the meta-analysis. The evidence suggests antenatal dietary and lifestyle intervention in obese pregnant women reduces maternal pregnancy weight gain (10 randomised clinical trials; n = 1228; -2.21 kg (95% confidence interval -2.86 kg to -1.59 kg)) and a trend towards a reduction in the prevalence of gestational diabetes (six randomised clinical trials; n = 1,011; odds ratio 0.80 (95% confidence interval 0.58 to 1.10)). There were no clear differences reported for other outcomes such as Caesarean delivery, large for gestational age, birth weight or macrosomia. All available studies were assessed to be of low to medium quality.

**Conclusion:**

Antenatal lifestyle intervention is associated with restricted gestational weight gain and a trend towards a reduced prevalence of gestational diabetes in the overweight and obese population. These findings need to be interpreted with caution as the available studies were of poor to medium quality.

## Background

Both developed and developing countries are experiencing a rapid increase in the prevalence of obesity [1-3]. In the UK, 24% of women of reproductive age are now obese (body mass index (BMI) equal or greater than 30 kg/m^2^) and the prevalence appears to be increasing [4]. Studies in UK women show that the rates of obesity in pregnancy have almost doubled in the last two decades [5,6]. Recent estimates suggest the prevalence of obesity in pregnancy in the UK is at least 20% with 5% having severe or morbid obesity [7,8].

Observational study data has linked obesity in pregnancy with adverse maternal and infant outcomes [7-10]. Obesity increases the risks of gestational diabetes [8,10-12], hypertensive disease (including pre-eclampsia) [8,13,14], thromboembolism [15,16], infection [14,17], Caesarean section [8,18], congenital fetal anomalies [19], macrosomia [13], induction [20], stillbirth [12], shoulder dystocia [14] and preterm delivery [21]. Moreover, maternal obesity may impact on long-term outcomes such as the increasing weight of the child in infancy and the severity of obesity in future generations [10,22,23].

As most of the adverse outcomes of obese pregnancies show strong associations with pre-pregnancy BMI, it is reasonable to assume that the ideal intervention would be to reduce obesity prior to pregnancy [24]. However, this is difficult to achieve because 50% of pregnancies in the UK are unplanned and a recent study concluded that only a small proportion of women planning pregnancy follow nutrition and lifestyle recommendations [25]. As such, an intervention pre-pregnancy may reach only a small proportion of the intended women.

Alternatively, pregnancy itself may represent an ideal opportunity to target lifestyle change as women have increased motivation to maximise their own health and that of their unborn child [25]. However, evidence of benefit from published intervention studies appears limited and inconsistent [26-44]. We therefore sought to determine the efficacy of combined dietary activity and behaviour support interventions in overweight and obese pregnant women by undertaking a systematic review and meta-analysis according to PRISMA (Transparent Reporting of Systematic Reviews and Meta-analyses) criteria for maternal clinical outcomes of weight gain, gestational diabetes and Caesarean section and infant outcomes, such as large for gestational age and macrosomia. Our aim was to generate data of the highest statistical power and sensitivity. Hence, in comparison with previous similar themed systematic reviews [45-48], we chose to interrogate multiple databases (not restricted to English) and also separately meta-analyse randomised clinical trials (RCTs) and non-RCTs evaluating relevant clinical outcomes, including gestational diabetes and Caesarean section, which had not been attempted in prior meta-analyses.

## Methods

### Eligibility criteria

The eligible studies included RCTs and non-RCTs that evaluated antenatal dietary and lifestyle interventions in obese and overweight pregnant women whose outcome measures included quantitative maternal and fetal health outcomes. Systematic reviews and trials of women with existing gestational diabetes, or trials of pre-conception or postpartum interventions, were not included. Inclusion of trials was not restricted by language, publication date or country. Systematic reviews and observational studies were excluded.

### Information sources

Literature searches were performed using five mainstream electronic databases (Cochrane Library, MEDLINE, EMBASE, CINAHL, Maternity and Infant care), and eight other databases (PsyclINFO via OVID SP, PyscLNFO via OVID SP, Science Citation Index via Web of Science, Social Science Citation Index via Web of Science, Global Health, Popline, Medcarib, Nutrition database).

### Search strategy

The following MeSH terms, words and combinations of words, were used in constructing the systematic search: overweight OR obesity; pregnancy OR pregnancy complications OR pregnancy outcome OR prenatal care, prenatal, antenatal, intervention, randomised controlled trial, life style, "early intervention (education)", health education, education, patient education handout, patient education, exercise, exercise therapy, health promotion, diet, carbohydrate-restricted, diet, fat-restricted, diet, reducing, diet therapy, weight loss. Full details of the search strategy are shown in Table 1. The searches were unlimited by time up to January 2012 and limited to human studies and clinical trials. The systematic search was undertaken in the mainstream databases and targeted searches were conducted in the other databases.

**Table 1:** Search strategy utilised for MEDLINE 1946 to January 2012

Batch	Search term (MESH)	Combination	Result
1	Pregnancy Complications/OR Pregnancy/OR Pregnancy Outcome/OR Pregnancy, High Risk/		646,055
2	Prenatal Care/OR Pregnancy/OR Pregnancy Complications		647,726
3	Antenatal.mp.		18,393
4	Gestation intervention.mp.		4
5		1 OR 2 OR 3 OR 4	651,321
6	Overweight.mp. OR Obesity/OR Overweight/OR Body Weight/		249,097
7	Obesity/OR Obesity, Morbid/or Obesity.mp.		145,882
8	Body Weight/OR Obesity/OR Body Mass Index/or BMI.mp. OR Overweight/		293,584
9		6 OR 7 OR 8	328,089
10		5 AND 9	21,583
11	Diet, Fat-Restricted/OR Diet/OR Diet, Protein-Restricted/OR Diet, Carbohydrate-Restricted/OR Diet.mp. OR Diet, Reducing/OR Diet Therapy/		255,985
12	Life Style/		36,837
13	Health Education/		48,625
14	Patient Education as Topic/		63,238
15	Exercise.mp. OR Exercise/OR Exercise, Therapy/		192,937
16	Health Promotion/		43,967
17	Weight Loss/		19,434
18		11 OR 12 OR 13 OR 14 OR 15 OR 16 OR 17	601,919
19		10 AND 18	3,769
20		LIMIT 19 TO ((female or humans or pregnancy) and (clinical trial, all OR clinical trial, phase i OR clinical trial, phase ii OR clinical trial, phase iii OR clinical trial, phase iv OR clinical trial OR controlled clinical trial OR randomized controlled trial))	**154**

### Study selection

Electronic literature searches, study selection, methodology, appropriateness for inclusion and quality appraisal were performed independently and in duplicate by two authors (E-ON and RV). Disagreements between reviewers were resolved by consensus. Included studies were divided into two groups (RCTs and non-RCTs) and separately meta-analysed.

### Data collection process

Two independent reviewers extracted the data. As a first step, each paper was screened using the title and the abstract. In the next round, studies were assessed for methodological quality and appropriateness for inclusion by two reviewers working independently from the full text of the manuscript. This was done without consideration of the results.

### Data items

For each included trial, data was extracted on maternal gestational weight gain; gestational diabetes; Caesarean section; large for gestational age baby (> 4 kg); and birth weight. The included studies have been summarised in Tables 2 and 3.

**Table 2:** A summary of the studies that met the criteria of the systematic review on lifestyle interventions in overweight and obese pregnant women: randomised trials

Author (year)	Ethnic group/Country	Participant/setting	Sample size	Intervention	Outcome measure(s)	Conclusion
Polley *et al. *(2002) [32]	31% black and 61% white/USA	Recruited before 20 weeks of pregnancy (normal BMI > 19.5 to 24.9; overweight BMI ≥ 25 to < 30 kg/m^2^)/Hospital based	120, including 49 overweight 59 in control arm; 61 in intervention arm	Exercise and nutrition information (oral and newsletter) Personalised graphs and behavioural counselling.	Gestational weight gain; gestational diabetes; Caesarean section; birthweight	No statistically significant reduction in gestational weight, prevalence of gestational diabetes, Caesarean section, or large for gestational age baby
Hui *et al. *(2006) [33]	Predominantly Caucasian/Canada	Less than 26 weeks pregnant (community based and antenatal clinics). All BMI categories. Mean BMI of non-intervention arm = 25.7 (SD = 6.3) and for intervention arm = 23.4(SD = 3.9)	45 21 in non-intervention arm; 24 in intervention arm	Physical exercise (group-sessions home-based exercise) Individualized nutrition plans	Gestational weight gain	No statistically significant reduction in gestational weight gain
Wolff *et al*., 2008 [30]	100% Caucasian/Denmark	Obese (BMI ≥ 30 kg/m^2^) women enrolled at 15 weeks' gestation	50 analysed 23 in control arm; 27 in intervention arm	Intensive intervention with 10 one-hour visits with a dietician at each antenatal visit, dietary guidance provided	Gestational weight gain; gestational diabetes; Caesarean section; birthweight	Statistically significant reduction in gestational weight gain, no statistically significant reduction in prevalence of gestational diabetes or Caesarean section, or birthweight
Jeffries *et al*., 2009 [28]	> 90% Caucasian/Australia	Women at or below 14 weeks' gestation. All BMI categories included	286 138 in control arm; 148 in intervention arm	Personalised weight measurement card (based on Institute of Medicine guidelines). Control had only single measurement at enrolment	Gestational weight gain	No statistically significant reduction in gestational weight gain.
Ong *et al*., 2009 [42]	Predominantly Caucasian/Australia	Pregnant obese women recruited at 18 weeks' gestation	12 six in control arm; six in intervention arm	Personalised 10 weeks of home-based supervised exercise (three sessions per week)	Maternal aerobic fitness and gestational diabetes	No statistically significant difference in aerobic fitness or gestational diabetes
Barakat *et al*., 2011 [41]	100% Caucasian/Spain	All BMI categories	160 80 in control arm; 80 in intervention arm	Three group-based sessions per week, light resistance and toning exercise from the second trimester	Gestational weight gain and birthweight	No statistically significant difference in gestational weight gain and birth weight. Exercise intervention might attenuate adverse consequences of maternal BMI on newborn birth size
Asbee *et al*., 2009 [27]	26% African American/USA	Pregnant women recruited before 16 weeks' gestation. All BMI categories except those of BMI > 40 kg/m^2^	100 43 in control arm; 53 in intervention arm	One session of dietetic counselling and activity	Gestational weight gain; pregnancy outcome	Statistically significant reduction in gestational weight gain. No effect on pregnancy outcome
Thornton *et al*., 2009 [29]	41% African American/USA	Obese pregnant women (BMI ≥ 30 kg/m^2^) recruited between 12 and 28 weeks' gestation	257 randomised. 25 lost to follow up. 116 in control arm; 116 in intervention arm	Nutritional regime for gestational diabetes	Gestational weight gain; gestational diabetes; Caesarean section; pregnancy outcome	Statistically significant reduction in gestational weight gain, no statistically significant reduction in prevalence of gestational diabetes, Caesarean section or birthweight
Guelinckx *et al*., 2010 [26]	100% Caucasian/Belgium	Obese (BMI > 30 kg/m^2^) women enrolled at 15 weeks' gestation.	195 randomised 85 analysed 65 in control arm; 65 in passive arm, 65 in intervention arm	Three arms: group sessions with a dietician; written brochures; and standard care Dietary and physical activity guidance provided by dietician and in written brochures	Nutritional habits; gestational weight gain; gestational diabetes; Caesarean section; birthweight	Improved nutritional habits; no statistically significant reduction in gestational weight gain, prevalence of gestational diabetes, Caesarean section or birthweight.
Phelan *et al*., 2011 [34]	67% White/USA	Pregnant women BMI between 19.8 and 40 kg/m^2 ^recruited between 10 and 16 weeks' gestation	401 randomised. 201 in non-intervention arm; 200 in intervention arm	Exercise and nutrition information (oral and newsletter) Personalised graphs and behavioural counselling	Gestational weight gain; gestational diabetes; Caesarean section; pregnancy outcome	Significant reduction in gestational weight gain; no statistically significant reduction in prevalence of gestational diabetes, Caesarean section or birthweight
Quinlivan *et al*., 2011 [59]	73% white, 19% Asian/Australia	Pregnant women: overweight (BMI 25 to 29.9 kg/m^2^) and obese (BMI ≥ 30 kg/m^2^)	132 randomised. 65 in non-intervention arm; 67 in intervention arm	Attended a study- specific antenatal clinic providing continuity of care, weighing on arrival, brief dietary intervention by food technologist and psychological assessment and intervention if indicated	Gestational weight gain; gestational diabetes; birthweight	Statistically significant reduction in gestational weight gain and prevalence of gestational weight gain. No statistically significant reduction in birthweight.
Luoto *et al*., 2011 [43]	Predominantly white/Finland	Pregnant women at risk of gestational diabetes. All BMI ranges	399 cluster randomised. 219 in non-intervention arm; 180 in intervention arm	Attended a study-specific individual antenatal lifestyle counselling clinic including group exercise	Gestational diabetes; gestational weight gain; birthweight	Statistically significant reduction in birthweight and macrosomia but no statistically significant difference in gestational diabetes
Nascimento *et al*., 2011 [44]	Predominantly white/Brazil	Pregnant women of all BMI categories	82 randomised. 42 in non-intervention arm; 40 in intervention arm	Attended a group-based exercise under supervision and received a home exercise counselling	Gestational weight gain; raised blood pressure; perinatal outcome	No statistically significant difference in gestational weight gain in terms of gestational weight gain, raised blood pressure or perinatal outcome

BMI: body mass index; SD: standard deviation

**Table 3:** Summary of the studies that met the criteria of the systematic review on lifestyle interventions in overweight and obese pregnant women: non-randomised trials

Author (year)	Ethnic group/country	Participants/setting	Sample size	Intervention	Outcome measure(s)	Conclusion
Gray-Donald *et al. *(2000) [38]	Native Americans/Canada	Recruited before the 26^th ^week of pregnancy, non-parallel recruitment of control and intervention arms. Mean BMI = 29.6 kg/m^2 ^(SD = 6.45) in non-intervention arm and mean BMI = 30.8 kg/m^2 ^(SD = 6.85) in intervention arm at baseline.	219 107 in non-intervention arm; 112 in intervention arm	Dietary and weight counselling Exercise groups provided	Gestational weight gain; gestational diabetes; Caesarean section; birthweight; postpartum weight retention	No statistically significant difference in gestational weight gain, prevalence of gestational diabetes, Caesarean section or large for gestational age baby
Olson *et al. *(2004) [51]	96% white/USA	Recruited before third trimester. Hospital and clinic setting BMI range: 19.8 to 29 kg/m^2^	498 381 in non-intervention arm; 117 in the intervention arm	Used the Institute of Medicine recommended guidelines on weight gain; 'health book' used to record diet and exercise and contained healthy eating and exercise information	Gestational weight gain; birthweight	No statistically significant reduction in gestational weight gain or prevalence of large for gestational age baby
Claesson *et al. *(2007) [36]	Not stated. Predominantly Caucasian/Sweden	Obese and registered at antenatal care clinic. BMI ≥ 30 kg/m^2^	348 193 in non-intervention arm; 155 in intervention arm	Nutritional habits interview, weekly counselling and aqua aerobic sessions	Gestational weight gain; Caesarean section.	Statistically significant reduction in gestational weight gain; no difference in prevalence of Caesarean section
Kinnunen *et al. *(2007) [37]	Over 90% Caucasian/Finland	First-time pregnant women who were obese (BMI ≥ 30 kg/m^2^)	196 95 in non-intervention arm; 101 in intervention arm	Individual counselling at each antenatal visits. Dietary guidance and optional activity sessions.	Gestational weight gain; diet change; birthweight	No statistically significant reduction in gestational weight gain or prevalence of large for gestational age baby. Statistically significant reduction in dietary glycaemic load.
Shirazian *et al*., 2010 [39]	33% blacks; 67% Latino/USA	Singleton obese (≥ 30 kg/m^2^) pregnant women recruited in the first trimester. Historical non-intervention group.	54 28 in non-parallel control arm; 28 in intervention arm)	One-to-one counselling; six structured seminars on healthy living (healthy eating and walking)	Gestational weight gain; gestational diabetes; Caesarean section	Statistically significant reduction in gestational weight gain; no difference in prevalence of gestational diabetes
Mottola *et al*., (2010) [35]	Not stated/Canada	Overweight (BMI ≥ 25 to 29.9 kg/m^2^) and obese (BMI ≥ 30 kg/m^2^) pregnant women recruited before 16 weeks' gestation; historical non-intervention group.	65 matched non-parallel control of 260	Individualised nutrition plan; exercise consisted of walking (three to four times per week, used pedometers)	Gestational weight gain; Caesarean section; birthweight; peripartum weight retention	Possible reduction in gestational weight gain; no difference in prevalence of Caesarean section or large for gestational age baby; minimal effect on peripartum weight retention

BMI: body mass index; SD: standard deviation.

### Risk of bias in individual studies

The quality of studies was assessed based on how the studies had minimised bias and error in their methods. We categorised the studies according to criteria based on PRISMA guidelines [49] and the Cochrane Library [50]. For example, high quality trials reported study aims; control comparison similar to the intervention group; relevant population demographics pre- and post-intervention; and data on each outcome. These study characteristics are tabulated in Tables 4 and 5. A final assessment categorised the studies as high, medium or low quality.

**Table 4:** Assessment of the quality of the included trials: non-randomised trials

Author (year)	Population representativeness	Adequacy of sequence generation	Masking/selection bias	Incomplete outcome data	Contamination	Sample size	Grade of quality
Gray-Donald *et al. *(2000) [38]	Yes: Registered from clinic	No	No	No	No: non-parallel control	219	Low
Olson *et al. *(2004) [51]	Yes	No	No	No	No: non-parallel control	560	Low
Claesson *et al. *(2007) [36]	Yes: Registered from clinic	No	No	Yes	No: selected from nearby city	315	Low
Kinnunen *et al. *(2007) [37]	Yes	No	No	No	Yes	55	Low
Shirazian *et al*., (2010) [39]	Yes	No	No	Yes	No: non-parallel control	28	Low
Mottola *et al*., (2010) [35]	Yes	No	No	Yes	No: non-parallel control	65	Low

**Table 5:** Assessment quality of included trials: randomised trials

Author (year)	Population representativeness	Adequacy of sequence generation	**Masking**/**selection bias**	Intention to treat	Incomplete outcome data	Loss to follow up	Sample size	Grade of quality
Polley *et al. *2002 [32]	Yes	Yes:	No	Not reported	No	Yes	120	Low
Hui *et al. *(2006) [33]	Yes: from clinic	Exact method not described	No	Not reported	No	Yes	52	Low
Wolff *et al*., 2008 [30]	Yes	Yes: computer generated	No	Not reported	Yes	Yes	50	Low
Jeffries *et al*., 2009 [28]	Yes	Yes: Opaque envelope	Yes	Not reported	Yes	Yes	286	Low
Ong *et al*., 2009 [42]	Yes	Exact method not described	No	Not reported	No	No	12	Low
Barakat *et al*., 2011 [41]	Yes	Yes	Yes	Yes	Yes	Yes	160	Medium
Asbee *et al. *2009 [27]	Yes	Yes	No	Not reported	Yes	No	100	Low
Thornton *et al*., 2009 [29]	Yes	Yes	Yes	Not reported	Yes	Yes	257	Medium
Guelinckx *et al*., 2010 [26]	Not reported	Randomised but not reported how	Not reported	Not reported	Yes	Not reported	99	Low
Phelan *et al*., 2011 [34]	Yes	Yes: Opaque envelope	Yes	Yes	Yes	Yes	401	Medium,
Quinlivan *et al*., 2011 [59]	Yes	Yes: Opaque envelope	Yes	Yes	Yes	Yes	124	Medium
Luoto *et al*., 2011 [43]	Yes	Yes	Yes	Yes	Yes	Yes	399	Medium
Nascimento *et al*., 2011 [44]	Yes	Yes: Opaque envelope	Yes	Yes	Yes	Yes	82	Low

### Summary and analysis of studies that meet the criteria

**Figure 1:**
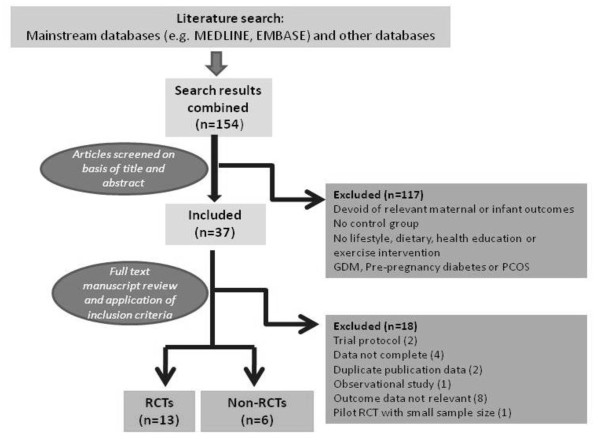
**Flow diagram of study selection**.

This is shown in Figure 1 and in a tabulated format contained within Table 2 and 3.

### Summary measures and data synthesis

The main measure of effect of the meta-analysis was the odds ratio or standardised mean difference. The data syntheses were conducted according to the Cochrane methodology [50]. First, we used statistical meta-analysis techniques to assess the efficacy of the interventions of controlled trials. Chi-square statistics tests were used to test for heterogeneity (Q statistics) between controlled trials. When there was no significant heterogeneity, we combined effect sizes in a fixed effect statistical meta-analysis using Review Manager (RevMan; Version 5.0, Copenhagen: The Nordic Cochrane Centre, The Cochrane Collaboration, 2008). The meta-analyses were performed by calculating the odds ratios (for proportion data) or standardised mean differences (for scale data) using a fixed effects model. Quantitative analysis was performed on an intention-to-treat basis focused on data derived from the period of follow-up. There was heterogeneity between studies because of the smaller sample size of some of the studies (poor quality), variation of the study population and the intensity and duration of the interventional strategies being evaluated. A random effects model was used to adjust for heterogeneity.

## Results

### Study characteristics

The review process is outlined in Figure 1 and the selected papers summarised in Tables 2 and 3.

Fifteen trials met the inclusion criteria: 13 RCTs [26-34] and six non-RCTs [35,36,38,39,51,52]. All 19 trials were performed in developed countries: five in the USA, three in Canada, three in Australia, two in Finland and one in Denmark, Netherlands, Sweden, Spain, Brazil and Belgium (Tables 2 and 3). Five RCTs were judged to be of medium quality [27,29,34]. The rest were deemed low quality (Tables 4 and 5).

The pooled RCTs included a total of 1,228 participants and the pooled non-RCTs included 1,534 participants. Participants were predominantly white except in the studies by Asbee *et al. *[27], Gray-Donald *et al. *[38] and Hui *et al. *[33]. In the Asbee *et al. *study, the majority were described as being of Hispanic ethnicity [27].

For all included RCTs, the control group received no intervention or standard care. In the non-RCTs, most used non-parallel controls [35,38,39,51] or controls from another centre [36]. The outcomes investigated in the trials were gestational weight gain, gestational diabetes, Caesarean section delivery, large for gestational age baby and birth weight.

### Effects of the intervention on outcomes

**Figure 2:**
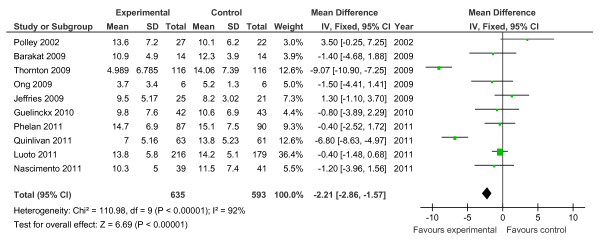
**Forest plot of randomised trials investigating the effect of lifestyle advice versus standard care on gestational weight gain (kg)**.

**Figure 3:**
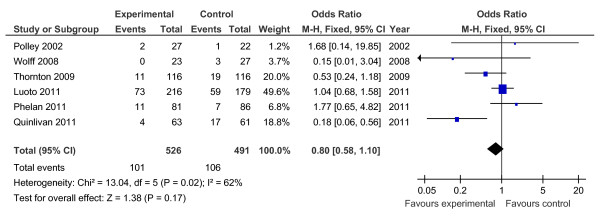
**Forest plot of randomised trials investigating the effect of lifestyle advice versus standard care on risk of gestational diabetes**.

**Figure 4:**
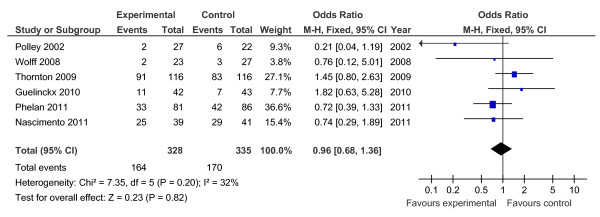
**Forest plot of randomised trials investigating the effect of lifestyle advice versus standard care on risk of Caesarean delivery**.

**Figure 5:**
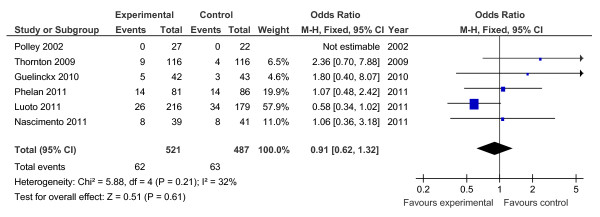
**Forest plot of randomised trials investigating the effect of lifestyle advice versus standard care on risk of large for gestational age baby**.

**Figure 6:**
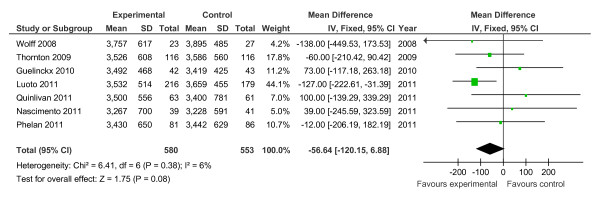
**Forest plot of randomised trials investigating the effect of lifestyle advice versus standard care on birthweight**.

**Figure 7:**
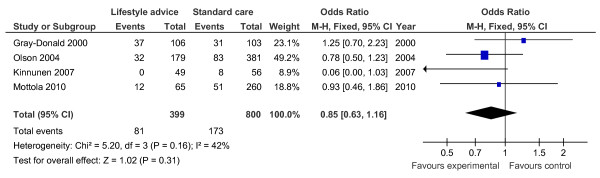
**Forest plot of non-randomised trials investigating the effect of lifestyle advice versus standard care on risk of large for gestational age baby**.

**Figure 8:**
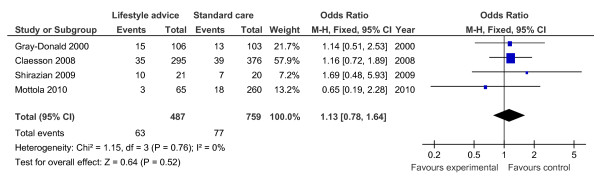
**Forest plot of non-randomised trials investigating the effect of lifestyle advice versus standard care on risk of Caesarean section**.

**Figure 9:**
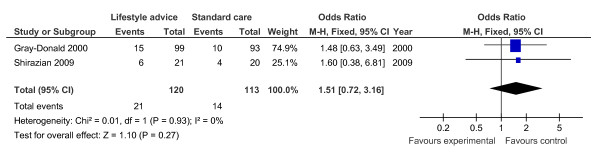
**Forest plot of non-randomised trials investigating the effect of lifestyle advice versus standard care on risk of gestational diabetes**.

**Figure 10:**
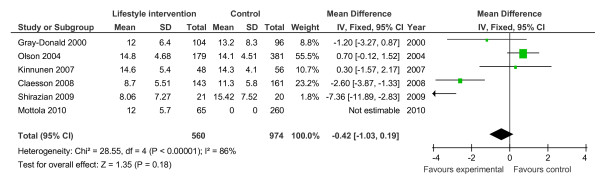
**Forest plot of non-randomised trials investigating the effect of lifestyle advice versus standard care on gestational weight gain (kg)**.

Of the 19 controlled trials, 16 measured gestational weight gain (10 randomised, 6 non-randomised); 8 recorded gestational diabetes (6 randomised, 2 non-randomised); 10 recorded Caesarean delivery (6 randomised, 4 non-randomised); 10 measured large for gestational age (6 randomised, 4 non-randomised); and 7 measured birth weight (7 randomised). Meta-analyses for the different outcomes are shown in Tables 6 and 7, and Figures 2, 3, 4, 5, 6, 7, 8, 9 and 10.

**Table 6:** Effect estimates for randomised trials of lifestyle advice versus standard care

Outcome or subgroup	Studies	Participants	Statistical method	Effect estimate
Large for gestational age	6	1,008	Odds ratio (Fixed, 95% CI)	0.91 (0.62, 1.32)
Caesarean delivery	6	663	Odds ratio (Fixed, 95% CI)	0.96 (0.68, 1.36)
Gestational diabetes	6	1,017	Odds ratio (M-H, Fixed, 95% CI)	0.80 (0.58, 1.10)^a^
Gestational weight gain (kg)	10	1,228	Mean difference (Fixed, 95% CI)	-2.21 (-2.86, -1.57)^a^
Birth weight (g)	7	1,133	Mean difference (Fixed, 95% CI)	-56.64 (-120.15, 6.88)

^a^Statistically significant pooled estimates. CI: confidence interval

**Table 7:** Effect estimates for non-randomised trials of lifestyle advice versus standard care

Outcome or subgroup	Studies	Participants	Statistical method	Effect estimate
Large for gestational age	4	1,199	Odds ratio (Fixed, 95% CI)	0.85 (0.63, 1.16)
Caesarean delivery	4	1,246	Odds ratio (Fixed, 95% CI)	1.13 (0.78, 1.64)
Gestational diabetes	2	233	Odds ratio (Fixed, 95% CI)	1.51 (0.72, 3.16)
Gestational weight gain (kg)	6	1,534	Mean difference (Fixed, 95% CI)	-0.42 (-1.03, 0.19)

CI: confidence interval

Meta-analysis of RCTs showed that combined antenatal lifestyle, dietary and activity intervention restricts gestational weight gain (Table 6 and Figure 2) and there was a trend towards reduction in the prevalence of gestational diabetes in overweight and obese women (Table 6 and Figure 3). However, meta-analysis of non-RCTs only showed weak evidence that lifestyle intervention reduces gestational weight gain (Table 7 and Figure 7) and there was no evidence for a reduction in prevalence of gestational diabetes (Table 7 and Figure 8). There was no robust evidence that lifestyle intervention is associated with a lower prevalence of Caesarean delivery or macrosomia or any alteration in birth weight (Tables 6 and 7, Figures 4,5, 6, 8, 9 and 10).

### Intervention characteristics

The nature of the interventions varied widely between studies and some of the key features of the interventions are outlined in Tables 2 and 3. In summary, for the six non-RCTs, three of the interventions comprised individual and group or seminar components [36,38,39,53], two were individual [35,52] and one was unclear [51]. Of the 13 RCTs, one comprised individual and group components [33], eight were individual [27-30,32,34] and three were group-based [26]. Where there were individual and group components, the latter were usually physical activity sessions. All of the non-RCTs included dietary and physical activity guidance, as did the majority of the randomised studies. Exceptions were two studies which included only nutritional guidance [29,30] and one which included guidelines about weight gain and weight monitoring only [28]. The majority of studies included dietary or physical activity guidance, with one of the non-RCTs [35] and three of the RCTs [29,32-34] specifying that guidance was personalised.

## Discussion

### Summary of main findings

Antenatal lifestyle, dietary and activity advice for overweight and obese pregnant women restricts maternal weight gain during pregnancy and lowers the prevalence of gestational diabetes in women who are overweight or obese. However, the quality of the study designs was generally poor. The reduction in gestational weight gain was observed to be statistically significant in the meta-analysis of randomised trials (10 RCTs; n = 1,228; -2.21 kg (95% CI, -2.86 to -1.57 kg)) but non-significant in the meta-analysis of non-randomised trials (six non-RCTs; n = 1,534). No effects of antenatal lifestyle interventions were identified in obese and overweight pregnant women in relation to Caesarean delivery, large for gestational age, birth weight and macrosomia (> 4 kg).

### Interpretation

There is evidence to suggest antenatal lifestyle interventions may restrict gestational weight gain and a trend towards a reduced prevalence of gestational diabetes, but there was no statistical effect on other important clinical outcomes, possibly due to inadequate power of the combined sample size. The effect on restricted weight gain and gestational diabetes was not consistent across all the trial populations and therefore cannot be generalised. There was also wide variation in the types of interventions evaluated in the studies. The majority were individual-based and most provided generic guidance comprising mainly dietary and physical activity information, with few tailoring guidelines. There was considerable heterogeneity in intervention design and no obvious patterns between intervention type and study outcomes. For the gestational weight gain and gestational diabetes outcomes, both the successful and non-successful studies included those which were personalised, combined physical activity and dietary guidance and were individual-based. Moreover, degrees of weight gain restriction achieved were modest overall. It is even harder to make conclusions regarding the specific behaviour change strategies included (for example, monitoring and goal setting) or theoretical basis of interventions since these were typically poorly reported.

Identifying specific components of successful interventions aids understanding of how interventions are having an effect and clear reporting of intervention design allows for easier replication [54]. Previous reviews have attempted to make conclusions regarding specific effective components of interventions. Suggestions that weight monitoring and setting weight goals could be useful [46] and also monitoring along with education counselling and physical activity sessions [51,55] have been made. Another review suggested that interventions should be based on the Theory of Planned Behaviour, but the rationale for using this model over others in this population was unclear [56]. None of these reviews examined intervention components systematically. A more recent review by Gardner *et al. *assessed interventions targeting gestational weight gain from a psychological perspective and specifically examined intervention content and delivery methods [57]. This review comprised 10 controlled trials, all included in the current review; only two of the studies reported basing interventions on theory and the studies used, on average, five behaviour change strategies (self-monitoring, feedback provision and setting behavioural goals were the most common), but no conclusions could be drawn as to their contribution to study outcomes. Broadly consistent with this were the four studies in the current review which were not included in the review by Gardner *et al. *[57]. Their review questioned the evidence supporting the benefits of weight monitoring, but tentatively suggested that information provision had been underused and that it might be of benefit to have a narrower focus of intervention targets [57].

### Comparison with other systematic reviews and strengths

Our study adds to a growing body of evidence that aims to evaluate lifestyle intervention as a means to minimise the adverse outcome associated with obesity in pregnancy. In comparison to other published reviews [45,46,56], we have adopted an original approach by broadening the literature source (multiple data sources, no language restriction), focusing on relevant clinical outcomes (such as Caesarean section, gestational diabetes, macrosomia), and improving our sensitivity by meta-analysing both RCTs and non-RCTs. Furthermore, to minimise bias, the review methodology was registered *a priori *(Prospero number CRD420111122 http://www.crd.york.ac.uk/PROSPERO). We therefore believe our review provides a comprehensive and reliable analysis of the current evidence and for the first time highlights that lifestyle intervention in pregnancy may reduce the prevalence of gestational diabetes.

### Limitations of this systematic review

The evidence summarised in this work comes from available studies of which most are of low quality, with only four studies fulfilling a medium quality score. Hence, the evidence base is weak and calls for more robust studies. Our trial population is relatively small, the intensity and duration of the interventions of trials varied and trials were predominantly USA in origin; a phenomenon common to many public health reviews, especially on obesity. Although our focus was on antenatal lifestyle intervention for obese and overweight pregnant women, our search yielded some studies that contained a mixed group of obese and normal weight women and we excluded all the non-obese participants from our analysis. Still, this may lead to inconsistencies in measuring the effect of the intervention as well as under- or overestimating the treatment effect. Furthermore, even though our search was systematic and rigorous, we could have missed eligible studies inadvertently.

## Conclusions

This review reveals that lifestyle interventions for obese and overweight women during pregnancy restrict gestational weight gain and a trend was evident towards reducing the prevalence of gestational diabetes. However, the quality of the published studies is mainly poor. This then highlights a paradox. At a time when solutions to address adverse outcome associated with maternal overweight and obesity are identified as a public health priority, we find that most of the research evidence lacks robustness to inform future evidence-based lifestyle interventions for obese pregnant women. There is thus a research gap regarding the effectiveness of lifestyle intervention in pregnancy. It is unlikely that further meta-analysis will help to refine the quality of evidence because studies demonstrated significant heterogeneity in relation to demography, outcome measurement, follow-up and degree of intervention. Hence, we conclude that there is the need for a well-designed large-scale prospective trial which examines combined antenatal lifestyle interventions in obese pregnant women that is suitably powered and incorporates robust methodology in accordance with standards set by Medical Research Council's framework for evaluating complex interventions [58]. There are two such studies which are currently ongoing called LIMIT (ACTRN 12607000161426) and UPBEAT (ISRCTN89971375). Both of these studies are appropriately powered to show convincingly whether lifestyle intervention is most likely to improve pregnancy outcome or not.

## Competing interests

The authors declare that they have no competing interests.

## Authors' contributions

The idea was conceived by PD and EO-N. The literature search and meta-analysis were by RV and EO-N and all contributed to the write up. All authors read and approved the final version of the manuscript.

## Pre-publication history

The pre-publication history for this paper can be accessed here:

http://www.biomedcentral.com/1741-7015/10/47/prepub
